# Affiliate Stigma Among Caregivers of Older People Living with HIV: A Descriptive Phenomenological Study

**DOI:** 10.3390/bs16060990

**Published:** 2026-06-15

**Authors:** Xiaohui Peng, Shan Wu, Liwen Jiang, Yanhua Chen, Fengling Dai

**Affiliations:** 1 School of Nursing, Southwest Medical University, Luzhou 646000, China; pxh19862550530@outlook.com (X.P.);; 2 Department of Science and Technology, Southwest Medical University, Luzhou 646000, China

**Keywords:** HIV/AIDS, older adults, caregivers, affiliate stigma, qualitative research

## Abstract

Background: The pivotal role of caregivers in HIV care for older people living with HIV (PLWH) stands in stark contrast to the scarcity of research on their experiences, particularly regarding affiliate stigma. Older PLWH face a unique intersection of HIV-related stigma and ageism, which may place their family caregivers at heightened risk of affiliate stigma. However, the manifestations, sources, and coping strategies related to this stigma remain poorly understood. Methods: The descriptive phenomenological study was conducted between May and June 2025 at an HIV care clinic of a tertiary hospital in Sichuan Province, China. Using purposive sampling, fifteen caregivers of elderly individuals living with HIV were recruited. Data were collected through face-to-face, semi-structured interviews. Results: Four overarching themes and eleven sub-themes were extracted: (1) sources of affiliate stigma—‘Inadequate knowledge of HIV transmission routes’, ‘Ageism’, and ‘Infidelity stigma’; (2) experiences of affiliate stigma—‘Stigma endorsement’, ‘Concealment of a family member’s HIV-positive status’ and ‘Psychological distress’; (3) consequences of affiliate stigma—‘Estrangement among family members’, ‘Substantial caregiver burden’ and ‘Social avoidance’; and (4) coping with affiliate stigma—‘Enhancing knowledge of HIV/AIDS’ and ‘Seeking social support’. Conclusion: This study investigates affiliate stigma among caregivers of older people with HIV. Healthcare providers should recognize this stigma and its negative effects. Effective interventions must be developed to alleviate this burden, thereby improving the welfare of both caregivers and patients.

## 1. Introduction

The aging global population, combined with advancements in antiretroviral therapy (ART), has led to a significant increase in the number of older individuals living with HIV. However, HIV-related stigma and ageism continue to pose substantial challenges (Guaraldi et al., 2024; Hsieh et al., 2022), AIDS-related stigma and ageism do not operate in isolation but intersect and synergize, creating a compound effect greater than the sum of their parts. This systemic oppression not only exacerbates detrimental impacts on the physical and mental health, quality of life, and social well-being of older adults living with HIV but also establishes ageism as potentially the most critical barrier to achieving healthy aging in this population. The World Health Organization defines older people living with HIV (PLWH) as individuals aged 50 years and above (Kiplagat et al., 2022). According to UNAIDS, the global population of older PLWH increased from 7.5 million in 2019 to 9.9 million in 2023 (UNAIDS, 2024). Similarly, data from China indicate that the proportion of older PLWH increased from 22% in 2011 to 44% in 2022 (Jin et al., 2023).

As the country with the world’s largest aging population, China has attracted increasing domestic and international attention to the aging of its HIV-positive population. In response, the 13th Five-Year Action Plan for AIDS Prevention and Control in China formally designated older adults as a priority population for HIV prevention and control. Although ART has significantly improved clinical outcomes (H. L. Tang et al., 2023), stigma against older PLWH remains prevalent (Fu et al., 2023). Particularly noteworthy in the Chinese context is the fact that sexual activity among older adults is often subject to cultural stigma (Hao et al., 2024), resulting in a “dual stigma” experienced by older PLWH—they face discrimination both due to their HIV status and for perceived deviations from age-related norms of sexual behavior.

This stigma not only directly affects patients but also extends to family caregivers through affiliate stigma (Meanley et al., 2019), exerting diverse negative impacts on their psychological well-being, family relationships, and quality of care (Yu et al., 2016). However, research on affiliate stigma among caregivers of older PLWH, particularly regarding its manifestations and influencing factors in the Chinese cultural context, remains limited. Therefore, it is essential to explore the experiences, sources, impacts, and coping mechanisms of stigma within this population to develop targeted interventions and promote healthy aging.

## 2. Background

Affiliate stigma refers to the phenomenon in which caregivers experience prejudice and discrimination due to their close association with the individual receiving care, often resulting in negative self-perception and emotional distress (Corrigan et al., 2006). The research focuses on the sources, impacts, and coping mechanisms of affiliate stigma.

Affiliate stigma does not stem from a single source. Instead, it arises from the intricate interplay of multi-level and multi-dimensional social and cultural factors. Within the community, misconceptions and fears about HIV transmission have led to caregivers being ostracized as “potential sources of infection (Kipp et al., 2007; Njuguna et al., 2023).” Concurrently, societal moral condemnation and stigmatizing labels have further exacerbated this stigma. HIV infection is often erroneously linked to immoral behavior, resulting in caregivers and their families being subjected to moral blame for “inadequate upbringing” or perceived misconduct (Li et al., 2008; J. Tang et al., 2024). In the family context, the home often becomes the most immediate and painful source of affiliate stigma. Exclusionary behaviors among relatives, such as refusing to share meals or verbal insults, not only intensify familial conflict but can even lead to caregivers being ostracized or expelled from the household (Atanuriba et al., 2021). Furthermore, the medical and support systems can inadvertently reinforce this stigma. For instance, specialized service protocols or aid supplies with prominent identifiers may inadvertently increase the risk of caregivers and patients being identified and singled out (Kipp et al., 2007; Tesfay et al., 2020).

Affiliate stigma exerts a series of profound negative impacts on caregivers, patients, and their families. On a societal level, the adage that “family skeletons should not be aired in public” compels families to actively conceal the illness and isolate themselves for fear of discrimination. This leads caregivers to sever social ties (Atanuriba et al., 2021; J. Tang et al., 2024). Consequently, the extended family, which should be a source of support, becomes one of exclusion and stress, resulting in the breakdown of social support systems (Atanuriba et al., 2021). Within the healthcare domain, the fear of exposure causes caregivers to conceal the illness, avoid medical care, interrupt treatment regimens, or decline external assistance (Busza et al., 2018; McHenry et al., 2017; Njuguna et al., 2023). This severely compromises treatment adherence and intervention efficacy (Tesfay et al., 2020). Economically, stigma can lead to job loss and the erosion of income (McHenry et al., 2017). Particularly vulnerable are women who, when ostracized by their families, are at greater risk of falling into poverty and shouldering the caregiving burden alone (Atanuriba et al., 2021). Furthermore, to avoid discrimination, caregivers may be forced to choose suboptimal educational environments for their children, thereby limiting the children’s developmental opportunities (Njuguna et al., 2023). Affiliate stigma thus damages individual mental health and weakens critical support networks. This, in turn, directly endangers patient treatment outcomes and overall quality of life.

Previous studies have proposed various coping strategies and intervention directions across individual, familial, communal, and systemic levels. On the individual and family levels, cognitive restructuring can help reframe AIDS as a manageable chronic disease, thereby adjusting one’s mindset (Kipp et al., 2007). Families should also proactively build support networks, strengthen internal cohesion, and seek help from peer groups (Li et al., 2008; McHenry et al., 2017). When necessary, concealment and confidentiality may serve as short-term coping mechanisms (Busza et al., 2018; McHenry et al., 2017). On the community and healthcare system levels, comprehensive health education is essential to correct misconceptions (Kipp et al., 2007; Njuguna et al., 2023). The risk of exposure can be reduced by integrating medical services, using discreet packaging for aid supplies, and offering remote consultations (McHenry et al., 2017; Tesfay et al., 2020). Additionally, community health workers should be trained to employ flexible and non-discriminatory approaches to foster trust. On the policy and structural levels, it is crucial to implement family-centered interventions that enhance family capabilities (Bogart et al., 2008; Li et al., 2008), provide economic support to reduce vulnerability (McHenry et al., 2017), and transform social attitudes through advocacy measures such as anti-discrimination legislation and mass media campaigns (Li et al., 2008).

In recent years, qualitative research on affiliate stigma experienced by caregivers of PLWH has garnered increasing global attention. Existing studies have primarily focused on specific demographic groups, such as children, adolescents, and female caregivers (Atanuriba et al., 2021; Njuguna et al., 2023; Pallangyo & Mayers, 2009), whereas the experiences of affiliate stigma among caregivers of older adults living with HIV remain significantly underexplored. Notably, research from eastern China indicates that up to 53% of HIV/AIDS caregivers experience stigma (Wu et al., 2015). This finding not only underscores the severity of the problem but also necessitates further study among high-risk vulnerable groups, particularly elderly caregivers. Furthermore, while age-related stigma and sex-related stigma have been documented as intersecting forms of discrimination affecting older PLWH themselves, how these intersecting stigmas shape the affiliate stigma experiences of their caregivers remains underexplored—particularly in cultural contexts such as China, where traditional values of filial piety and the concept of ‘face’ may uniquely influence stigma processes (Yao et al., 2025). The present study addresses this gap by providing an in-depth qualitative exploration of affiliate stigma among caregivers of older PLWH in China, using a descriptive phenomenological approach to capture the lived experiences, sources, impacts, and coping strategies of this understudied population.

## 3. The Study

### 3.1. Aim

The aim of this study was to explore, through qualitative research, affiliate stigma among caregivers of older PLWH in southwestern China, to inform the development of targeted interventions, the design of tailored health education programs, and the optimization of the caregiver support system.

### 3.2. Design

Descriptive phenomenological research focuses on the primary conscious experiences of subjects and is therefore also referred to as the phenomenology of consciousness (Husserl, 1970). This approach emphasizes the description of lived experiences as they are directly given, abstaining from interpretive overlay, and aims to portray the real world from an objective and detached perspective. Guided by this framework, this study employed a descriptive phenomenological design to explore the experiences of affiliate stigma among caregivers of older PLWH. To achieve this aim, the following research question was formulated: “What are the lived experiences of affiliate stigma among caregivers of older PLWH?” This question is designed to elucidate both the “what” and “how” of the phenomenon, making it well-suited to a systematic investigation of the experiences and processes of affiliate stigma within this population. Furthermore, the study rigorously adhered to the reporting standards outlined in the Consolidated Criteria for Reporting Qualitative Research (COREQ) (Tong et al., 2007) to ensure methodological transparency and credibility.

### 3.3. Participants

This study was conducted in May–June 2025 at a dedicated HIV outpatient clinic of a tertiary hospital in Sichuan Province, China, focusing on caregivers of older PLWH. The selection of Sichuan as the study site is epidemiologically significant, as the province has reported the highest number of PLWH in China over the past two years (NCAIDS, 2024, 2025).

### 3.4. Inclusion and Exclusion Criteria

Participants were recruited through purposive sampling from family members who accompanied patients to medical visits and who also served as the primary caregivers in the home setting. Inclusion criteria required participants to be: (1) informal caregivers (e.g., family members, friends, or non-relatives) who assumed responsibility for the majority of daily care and were aware of the patient’s HIV status (Alam et al., 2020); (2) not diagnosed with HIV/AIDS; (3) aged 18 years or older; (4) capable of communicating clearly in Chinese; and (5) willing to provide informed consent. Individuals with psychological disorders were excluded.

### 3.5. Interview Guide 

This study adopted a phenomenological approach, which was implemented through a systematic process. Initially, the research team conducted a comprehensive review of existing literature on affiliate stigma and sought guidance from experts in the fields of HIV prevention and psychology. Building upon this foundation and informed by Pachankis’ cognitive–affective–behavioral model of concealable stigma (Pachankis, 2007), a semi-structured interview guide was developed for caregivers of older PLWH. The complete interview guide is presented in Table 1.

### 3.6. Data Collection

Data collection was carried out through semi-structured individual interviews conducted at a specialized care clinic within a tertiary hospital in Sichuan Province. Through purposive sampling, participants were recruited during periods when caregivers accompanied patients to their medical appointments. Prior to the study, the research team underwent comprehensive training in qualitative research methodologies and honed their interviewing techniques through simulated practice sessions. All participants completed the interview process in full, with no instances of hesitation or withdrawal. To ensure objectivity, interviewers had no prior relationships with participants, and no incentives were offered for participation.

The study strictly followed ethical principles regarding informed consent. Before each interview, participants were fully informed about the study’s purpose, procedures, potential benefits, and associated risks. Interviews were conducted in private settings to ensure confidentiality and lasted between 30 and 60 min.

With participants’ consent, all interviews were audio-recorded. Additionally, a second researcher documented non-verbal behaviors, such as body language, facial expressions, and changes in demeanor. Throughout the interviews, interviewers maintained a neutral posture, avoiding leading questions, suggestions, or interruptions to ensure the integrity and impartiality of the collected data.

### 3.7. Data Analysis

Data collection and analysis were conducted concurrently. All interviews were conducted in Mandarin by researchers fluent in the local dialect, who provided translation and cross-verification as necessary. Audio recordings were transcribed on the same day. Data were analyzed using Colaizzi’s seven-step phenomenological analysis method (Colaizzi, 1978), which included the following procedures:

(1) All interview transcripts were repeatedly reviewed multiple times to ensure thorough familiarity, with particular attention directed toward statements and underlying attitudes associated with affiliate stigma.
(2) Relevant statements concerning affiliate stigma were extracted, with a focus on those reflecting caregivers’ lived experiences of stigma.
(3) The extracted statements were coded to identify key insights and overarching themes related to the experiences, sources, impacts, and coping strategies associated with affiliate stigma.
(4) These coded insights were then synthesized into coherent thematic clusters, such as ‘Experiences of Affiliate Stigma’ and ‘Sources of Affiliate Stigma.’
(5) Each theme was further elaborated using caregivers’ own descriptions to illustrate how affiliate stigma manifested in their daily caregiving practices, social interactions, and psychological well-being.
(6) A comprehensive structure representing the core phenomenon of affiliate stigma was developed.
(7) Caregivers were contacted via telephone or WeChat to validate whether the identified themes accurately captured their experiences of affiliate stigma. Of the 15 participants, 13 were successfully contacted for member checking. Among them, 12 confirmed that the extracted themes accurately reflected their real experiences, while one participant was unable to complete the full check due to time constraints. None of the participants who completed the member checking raised any disagreement with the themes. Therefore, no themes were modified as a result.

**Figure 1:**
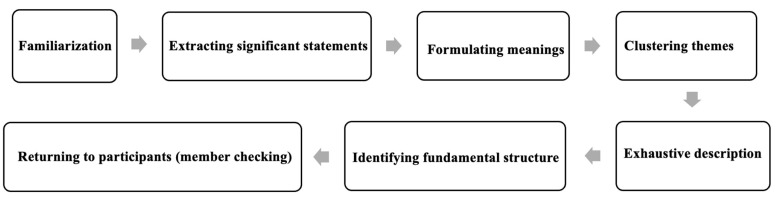
Colaizzi’s seven-step phenomenological analysis process as applied in this study. Adapted from Colaizzi (1978).

Figure 1 presents a schematic representation of Colaizzi’s seven-step phenomenological analysis process as applied in this study.

Participant recruitment was terminated upon reaching data saturation, defined as the point at which no new themes or codes emerged during the analytical process (Bowen, 2008).

### 3.8. Rigor

To ensure methodological rigor, this study followed the quality criteria framework proposed by Guba and Lincoln (1994). Rigor was established across four dimensions:

Credibility. To ensure that the findings accurately reflect participants’ actual experiences, researcher triangulation was employed during data analysis. Two researchers independently coded the data (kappa = 0.84); disagreements were resolved through discussion, with a third researcher consulted when necessary. Preliminary findings were returned to three participants for member checking, who confirmed that the results aligned with their experiences.

Transferability. To facilitate readers’ assessment of the applicability of the findings, detailed demographic characteristics of participants are presented in Table 2, and the research context (the designated HIV clinic in Zigong, local HIV epidemiology) is described.

Dependability. To ensure a transparent and traceable research process, original interview transcripts, coding records, analytical memos, and documentation of theme revisions were preserved. Negotiation processes for coding disagreements were recorded in detail, forming a complete audit trail.

Confirmability. To ensure that the findings emerged from the data rather than from researcher subjectivity, reflexive journals were maintained to document the researchers’ thoughts throughout the study, including potential cognitive biases arising from their professional backgrounds (nursing) and prior experience in HIV-related research. Original interview transcripts, coding records, and analytical memos were preserved to ensure that the reasoning pathway from raw data to research conclusions remained clear and auditable.

### 3.9. Ethical Considerations

Prior to the initiation of this study, all researchers had completed training in qualitative research methods and obtained ethical approval from the Ethics Committee of the Affiliated Hospital of Southwest Medical University (Approval No. KY2025235). The study strictly adhered to the ethical principles outlined in the Declaration of Helsinki (World Medical Association, 2013). The research team introduced themselves to the physicians and nurses at the Care Clinic, clearly explained the purpose of the study, and obtained verbal informed consent from all participants before conducting the interviews. Audio-recorded interviews were then conducted. The entire process upheld the principle of voluntary participation, and participants were informed of their right to withdraw from the study at any time without consequence.

## 4. Results

### 4.1. Study Participants

Fifteen caregivers, aged 27 to 75 and experienced in caregiving, participated in the study. The demographic characteristics of the participants are detailed in Table 2.

### 4.2. Major Themes

**Figure 2:**
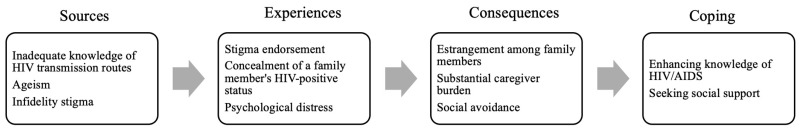
Schematic diagram of affiliate stigma among caregivers of older people living with HIV.

This study identified four themes and eleven sub-themes through a qualitative analysis of caregivers’ experiences with affiliate stigma. The key aspects of these experiences—including their sources, experiences, impacts, and coping managements—are summarized in Table 3. The logical relationships among these themes are illustrated in Figure 2.

### 4.3. Theme 1: Sources of Affiliate Stigma

#### 4.3.1. Inadequate Knowledge of HIV Transmission Routes

Some caregivers demonstrated a relatively limited understanding of HIV and lacked a comprehensive, scientifically grounded perspective on the disease. Their knowledge regarding transmission routes was often inadequate, leading to unwarranted caution and anxiety in the context of everyday interactions. This heightened sense of protection and misunderstanding, rooted in insufficient awareness, emerged as a significant contributor to affiliate stigma among caregivers.

*“I kept reminding my son and my little sister—don‘t ever use their bowls or towels.”*

(A1)

*“Now I stay out of the kids’ way—I don’t really get close to them anymore. She’s got her own bathroom and stuff. Like when I cook, I just dish out some food into her bowl and that’s all.”*

(A5)

*“Back then, they didn’t have much going on. They got a placenta from the midwife and just ate it … Maybe that’s how things went down.”*

(A14)

#### 4.3.2. Ageism

Some caregivers expressed astonishment that the patients had contracted AIDS in old age, suggesting an underlying perception that the elderly should not be sexually active.

*“I never thought my grandpa would end up with a sickness like this at his age … It’s not like he did anything wild or risky—he’s always been really careful with how he lived.”*

(A3)

*“My kids were pretty shocked at first too. They couldn’t stop asking how someone his age could even catch a disease like this.”*

(A9)

*“It’s been ten years, and I still haven’t said a word to my husband. My dad’s gotten so old lately—I just worry it would really hurt him if he ever found out.”*

(A13)

#### 4.3.3. Infidelity Stigma

Some caregivers reduced their affiliate stigma by acquiring HIV prevention knowledge, implementing standardized care practices, and educating family members about protection methods—a process that enhanced their coping efficacy.

*“He knew what he was doing—he understood the risks—and he still did it anyway. The longer I took care of him, the more I started to feel … like he’d let me down.”*

(A4)

*“He got it while he was working away from home. When we found out, I was totally heartbroken. He did something that really hurt me, and after that, we just kept fighting all the time.”*

(A7)

*“When the test results came back, I was absolutely furious. He always acted like such a decent, honest guy at home … But part of me has never really forgiven him.”*

(A15)

### 4.4. Theme 2: Experiences of Affiliate Stigma

#### 4.4.1. Stigma Endorsement 

Within specific sociocultural contexts, caregivers may gradually internalize the discriminatory attitudes prevalent in society toward individuals living with HIV, thereby developing internalized stigma. This psychological process involves the unconscious adoption and acceptance of such negative societal perceptions, ultimately leading to reduced self-worth and self-critical beliefs among caregivers.

*“Every time I went into that clinic … I felt weird, like people were judging me. It felt like everyone was staring at me like I didn’t belong.”*

(A5)

*“It was just … something I didn’t want to talk about. You know how people in the countryside can be—they really judge stuff like that. So only my brothers and sisters knew. We kept it quiet from everyone else.”*

(A9)

*“Around here, if people found out, they’d see it as some kind of moral problem. They’d probably cut us off. Honestly, nobody wanted to get mixed up in it.”*

(A14)

#### 4.4.2. Concealment of a Family Member’s HIV-Positive Status

Some caregivers chose to withhold the patient’s diagnosis due to concerns such as self-protection, the desire to avoid placing additional burdens on the family, and the intention to preserve the patient’s dignity, as they were apprehensive about the potential negative consequences of disclosure.

*“I told my family the test showed a tumor … I was too scared to say it was HIV-related. My girlfriend tried to look through my bag for the report, but I wouldn’t let her see it … Honestly, if I had told her, we might’ve broken up.”*

(A1)

*“My in-laws still don’t know … I was worried it would change how they see me. Whenever family stopped by, we just told them it was something with the lungs and back.”*

(A8)

*“I never told my mom … I was scared she’d see my dad differently. You know how it is—people treat it differently when a guy gets HIV.”*

(A9)

#### 4.4.3. Psychological Distress

In China, HIV often triggers an immediate fear response and strong social stigma. Caregivers of affected individuals frequently experience affiliate stigma, which manifests as intense anxiety, fear, sadness, and anger stemming from their association with the condition.

Anxiety among interviewees arose not only from the prospect of violating traditional customs upon their parents’ death from AIDS but also from bearing the disease’s heavy stigma.

*“Where I’m from, when someone passes away, you have to state clearly what illness they died from. Just thinking that one day my mom and dad will be gone, I really don‘t know what I’ll do then.”*

(A1)

*“At first, when I thought about this (my mom having AIDS), I didn’t sleep a wink all night … It felt like the world was ending.”*

(A5)

*“At the very beginning, I just couldn’t accept it at all (my dad having AIDS). I was up the whole night …”*

(A10)

For some interviewees, profound concern stemmed from the fear that a disclosure could trigger social stigma, rupture close relationships, hinder professional advancement, and tarnish their family’s standing.

*“I’m really worried people might find out—especially my friends. I’m seeing someone right now, not married yet … And honestly, my biggest fear is that if she finds out, she’ll dump me.”*

(A1)

*“If this ever got out, it’d completely ruin my career. I might even have to move out of this city.”*

(A4)

*“I’m worried people will get the wrong impression and start treating you differently … They act like this disease is something shameful—like just talking to you could give them the illness. My dad’s already pretty old. I really don’t want my parents or my whole family getting judged like that.”*

(A8)

Some interviewees described being overwhelmed by grief, stemming from an inability to accept their relatives’ AIDS diagnosis and the torment of feeling powerless to change it.

*“A lot of the time, I’ll just get hit with this really sad feeling outta nowhere … It just really gets to me.” (starts crying)*

(A1)

*“I’ve always thought of myself as pretty open-minded—but I just can’t wrap my head around this. I keep wondering, how could this even happen to my mom?”*

(A5)

*“Sometimes I just can’t help feeling upset … I end up thinking, why did he have to get stuck with this illness?”*

(A10)

For some interviewees, anger stemmed from the emotional dysregulation caused by the strains of caregiving. They released their pent-up frustration and helplessness through outbursts directed at relatives, only to be met with subsequent guilt or remorse.

*“I was just on the phone with my mom, and I kinda snapped at her … I might’ve been a bit too harsh. I told her, Just send it over already, stop making things so complicated.”*

(A1)

*“Sometimes, with all the stress from life and everything … when my dad and I don’t see eye to eye, I just end up snapping at him.”*

(A8)

### 4.5. Theme 3: Consequences of Affiliate Stigma

#### 4.5.1. Estrangement Among Family Members

Motivated by misconceptions about HIV or experiences of stigma, some caregivers adopted overly protective behaviors—such as eating separately or sleeping in different rooms. Although driven by concern, these behaviors undermined trust within the family, created emotional distance, and disrupted traditional family roles. In the long run, they contributed to a maladaptive reorganization of familial relationships.

*“When my dad tried to hold the baby, I’d jump in and stop him … I couldn‘t explain how I felt.”*

(A1)

*“Back then, my mom used her own bathroom and ate off her own plates. When I cooked, I’d just give her a bit of food in her bowl and that was all. But it wasn’t easy on her either—she got down when she was in the hospital.”*

(A5)

*“My dad had this condition … It was kind of hard to talk about, especially since my youngest was only eight at the time. He also had some dental problems, and I was worried his mouth might affect the kids. So we ended up living apart back then.”*

(A8)

#### 4.5.2. Substantial Caregiver Burden

Caregivers experienced prolonged dual stress from both societal stigma and caregiving responsibilities—a concealed psychological burden that contributed to enduring physical and emotional fatigue. On one hand, they faced affiliate stigma stemming from negative societal attitudes toward HIV. On the other hand, concerns about discrimination often discouraged them from delegating caregiving tasks to other family members, leaving many feeling isolated and emotionally depleted.

*“After all those years of looking after everyone … It wore me out, completely wiped me out, both physically and mentally.”*

(A1)

*“There are only two of us daughters. My older sister didn’t get much of an education, and she’s got her own family to look after, so we never told her about Dad’s illness. Now Mom’s getting older too, and I’m always worried about what’ll happen if either her or me gets sick down the line.”*

(A4)

*“This illness isn’t like other ones—it really took a toll on my emotions. I stayed up so many nights just thinking about everything.”*

(A5)

#### 4.5.3. Social Avoidance

Influenced by ‘face culture’, some caregivers actively engaged in social avoidance to prevent the potential disclosure of the patient’s HIV status. The primary motivation behind this protective strategy was to avoid possible social discrimination that could arise if the illness became publicly known.

*“I stopped going to hangouts with friends—I was just too scared someone would find out … Even when I took the kids out, I had to prepare all the food in advance. I couldn’t let her cook—I was always worried she might hurt herself.”*

(A5)

*“For the past six or seven years, I hardly hung out with them at all. I was scared they’d find out about my dad’s HIV.”*

(A8)

*“I couldn’t go out much because I had to take care of him. And to be honest, I was too embarrassed to go out anyway—I was always worried people would start asking me questions.”*

(A10)

### 4.6. Theme 4: Coping with Affiliate Stigma

#### 4.6.1. Enhancing Knowledge of HIV/AIDS

Caregivers often transitioned from heightened anxiety and moralistic evaluations to a more scientifically grounded understanding of HIV. With increasing caregiving experience, their perception of the illness became more accurate, resulting in a corresponding decrease in affiliate stigma.

*“But now that it’s part of my own life, and after caring for him for a while … It doesn’t feel scary anymore—it’s just normal now.”*

(A8)

*“At first, I was totally shocked—everyone kept saying it was some super dangerous, scary illness. But after a bit, I started realizing it’s really not that bad after all.”*

(A9)

*“I took the time to learn about this disease, so now I know what to look out for. Things like hugging, sharing meals, or just being around someone every day—none of that spreads the virus.”*

(A10)

#### 4.6.2. Seeking Social Support

In response to affiliate stigma, some caregivers employed proactive coping strategies, such as deliberately accepting their situation and seeking diverse forms of social support—including assistance from family and friends, access to medical resources, and government aid—to help ease family burdens.

*“We worked closely together and asked the medical staff for support whenever we needed it. Without them, we wouldn’t have gotten through everything step by step.”*

(A4)

*“I ended up telling my aunt and cousin—you know, the ones we’re really close with. Since we hang out at each other’s houses a lot and I sometimes need their support, I felt like I had to be honest about Dad’s situation.”*

(A10)

*“I was actually heading out this afternoon to apply for low-income benefits for him. I thought people in our town probably wouldn’t hear about it. I was a bit nervous at first, but the coverage is way better with those benefits.”*

(A13)

## 5. Discussion

Grounded in the cognitive–affective–behavioral model of concealed stigma, this study provides a qualitative exploration of affiliate stigma among caregivers of older PLWH, with a specific focus on their lived experiences of stigma, its impacts, sources, and managements. This qualitative study investigates affiliate stigma among family caregivers of older (aged 50 years or above) people living with HIV in China using a descriptive phenomenological approach.

The interviews revealed that caregivers of older PLWH commonly exhibited internalized stigma and various negative emotions. This finding is consistent with a phenomenological study conducted in Ethiopia, which reported that caregivers, due to their close association with PLWH, may experience discrimination and social exclusion even in the absence of personal infection (Alemu et al., 2013). The emotional impacts observed in our study align with those identified in previous research. For instance, a study from Ghana indicated that family caregivers of PLWH often experience psychological distress—such as anxiety and insomnia—because of affiliate stigma (Atanuriba et al., 2021). Our findings further demonstrate that the stigma and negative emotions experienced by caregivers of older PLWH are particularly pronounced, highlighting the urgent need for increased attention from healthcare providers, community-based HIV service organizations, and relevant institutions. This study provides a theoretical foundation for the development of interventions aimed at mitigating affiliate stigma in this population, thereby contributing to improved family harmony and psychological well-being.

This study identified two key sources of stigma among caregivers of older PLWH: inadequate disease awareness and ageism. These stigmatizing attitudes originate not only from public misconceptions about HIV transmission and prejudices toward older populations but are also internalized by older PLWH and their caregivers, resulting in self-stigmatization. The role of inadequate disease knowledge aligns with findings from previous studies; for example, in Kenya, limited public understanding of HIV transmission routes often leads to fear and misunderstanding toward caregivers within their communities (McHenry et al., 2017). This study provides new evidence that caregivers of older PLWH face dual stigmatization—encompassing both HIV-related social stigma and age-based discrimination directed at the older patients (Brown & Adeagbo, 2021; Guaraldi et al., 2024). Furthermore, while HIV-related stigma significantly disrupts the social networks of older patients and their families (Qiao et al., 2019), ageism further limits their access to essential social support from relatives and friends (Schrimshaw & Siegel, 2003). A two-pronged intervention approach is recommended. At the public education level, collaboration among healthcare institutions, community organizations, and elderly service agencies should be strengthened to implement systematic, science-based health communication campaigns aimed at improving societal knowledge of HIV and challenging age-associated stereotypes. At the individual level, structured psychological interventions are needed to help caregivers recognize and address internalized stigma, and to foster positive self-identity through established psychosocial mechanisms.

This study identified three sources of stigma affecting caregivers of older PLWH: HIV-related stigma, ageism, and infidelity stigma. Consistent with stigma theories, these stigmas do not operate independently but may interact to create synergistic effects. Stigma synergy occurs through two pathways. First, additive amplification: each stigma contributes independently to affiliate stigma, and their co-occurrence produces a compounded burden. Second, qualitative transformation: the convergence of multiple stigmas generates new stigma content absent in any single form. For example, caregivers may perceive their parent as both immoral (due to infidelity) and a burden (due to age-related frailty). This synergy may produce unique effects on caregivers. One is double concealment: caregivers must hide both the patient’s HIV status and age-related decline (e.g., frailty, frequent clinic visits). Another is anticipatory shame: caregivers may experience anxiety before events where disclosure could be required (e.g., hospital visits, funeral customs). These effects may delay help-seeking more severely than a single stigma alone, consistent with Pachankis’s (2007) cognitive–affective–behavioral model of concealable stigma.

The interview results indicated that long-term caregivers of patients were significantly affected by HIV-related affiliate stigma, with these impacts primarily manifesting in family relationships and caregiving burden. This study found that caregivers of older PLWH exhibited caregiving behavior patterns similar to those of caregivers of HIV-infected children, both showing noticeable attitude shifts following the patient’s diagnosis (Busza et al., 2018). Unlike caregivers of HIV-infected children, the caregiving context for older PLWH exhibits several distinctive features. First, while caregivers of HIV-infected children focus on protecting the child’s innocence, caregivers of older PLWH must confront the patient’s past behaviors (e.g., infidelity). This retrospective moral judgment—absent in child caregiving—can intensify feelings of betrayal, as reflected in the sub-theme of infidelity stigma. Second, the patient’s age introduces unique end-of-life concerns, such as funeral customs requiring disclosure of the cause of death—a source of anxiety specific to older adult caregiving. Third, filial piety operates differently when the patient is a parent versus a child. Traditionally, filial piety obligates adult children to care for aging parents as a debt of upbringing (Nie, 2021). However, this duty can paradoxically exacerbate affiliate stigma. As Qin argues, filial piety can function as both a resource and a hindrance, translating into requirements for secrecy—a burden intensified when the illness carries moral stigma. In contrast, when caring for an HIV-infected child, filial piety is less salient; stigma centers on the child’s innocence rather than moral failure.

In coping with affiliate stigma, caregivers employed diverse strategies. Consistent with previous studies, some caregivers resorted to concealing the patient’s diagnosis—a passive coping mechanism driven by two key factors: first, intense self-stigmatization led to the belief that disclosure would result in discrimination against the entire family (Larki et al., 2020); second, a strong motivation to protect the family’s dignity and preserve personal reputation prompted them to adopt concealment as a primary strategy (Bogart et al., 2008). Additionally, some caregivers proactively engaged in social avoidance due to fears that disclosure would trigger societal discrimination (Tesfay et al., 2020). It is worth noting that this study also found that caregivers who reduced their own social interactions often simultaneously restricted the patients’ social activities, ultimately leading to a contraction of the family’s overall social support network. On the other hand, some caregivers adopted active coping strategies, such as confiding in trusted relatives and friends and seeking professional support from healthcare providers, which effectively alleviated their experience of affiliate stigma (Alemu et al., 2013; Atanuriba et al., 2021). Furthermore, this study identified a similarity between caregivers of older PLWH and those caring for patients with colon cancer: both groups actively acquired disease-related knowledge to enhance their health literacy, which also served as an effective strategy for reducing affiliate stigma (Çakir et al., 2021). Meanwhile, evidence from systematic reviews suggests that audiovisual interventions delivered through public media channels by some governments have been effective in reducing HIV-related stigma and improving public attitudes and behaviors toward AIDS (J. Tang et al., 2024).

In this study, the affiliate stigma experienced by caregivers of older PLWH was evident across the cognitive, emotional, and behavioral dimensions of the cognitive–affective–behavioral model of concealed stigma. However, its manifestation in the self-evaluation dimension was not statistically significant. This phenomenon may be associated with the caregivers’ life stage and social roles. Caregivers in this age group often maintain a strong sense of purpose within society: unmarried individuals typically exhibit a deep sense of responsibility toward the older patients; those with children undertake the mission of guiding and nurturing the next generation; while spousal caregivers, often with lower levels of education and residing in rural areas, may be less aware of or concerned with the stigma associated with HIV, which could serve as a buffer against negative effects on their self-evaluation.

### 5.1. Limitations

This study has two main limitations. First, all participants were recruited from Sichuan Province, a region with a high HIV prevalence, and the majority of caregivers enrolled had relatively low socioeconomic status and educational attainment. These factors collectively limit the generalizability of the findings, which may not accurately reflect the experiences of caregivers in other regions of China. Future studies should include caregivers of older PLWH from diverse ethnic backgrounds, geographic regions, and healthcare settings to obtain more representative and comprehensive results. Second, although the research team was familiar with the local dialect, certain dialect-specific expressions and colloquialisms used by some older respondents may have introduced interpretation biases during transcription and translation, potentially affecting the accuracy of the data.

### 5.2. Future Lines of Research

Caregivers of older PLWH widely face stigma-related psychological distress and an increasing caregiving burden. This situation highlights a critical contradiction: even if the global 95-95-95 targets are achieved, current healthcare systems—particularly those in resource-limited settings—are still inadequately prepared to meet the growing care needs of this population (Kiplagat et al., 2022). Future policy efforts should focus on two key areas: first, integrating mental health screening for caregivers into national HIV prevention and care programs; and second, developing age-friendly support tools to address gaps in community-based care networks within the healthcare system.

## 6. Conclusions

This study revealed the complex phenomenon of affiliate stigma experienced by family caregivers of older PLWH within the Chinese cultural context, which was characterized by several key findings. First, caregivers commonly faced dual stigma—encompassing both HIV-related discrimination and ageism—that manifested not only as external social exclusion but was also internalized as self-stigmatization and negative emotional responses. Second, the culturally specific value of filial piety exerted a dual influence on caregiving behaviors: while it strengthened children’s sense of responsibility and promoted shared caregiving among family members across genders, the traditional belief that “family shame should not be made public” further reinforced illness concealment. Furthermore, caregivers exhibited distinct patterns across cognitive, emotional, and behavioral dimensions, including misconceptions about HIV transmission, conflicted emotional experiences, and a divergence into both adaptive and maladaptive coping strategies. Additionally, the study identified significant gaps in the current healthcare system, particularly the lack of mental health services and age-appropriate support tools tailored for caregivers of older PLWH. These findings provide a foundation for the development of culturally sensitive interventions, suggesting a need for anti-stigma initiatives that incorporate filial piety values, establish multi-level support networks for caregivers, and integrate mental health screening into standard HIV care programs to improve the quality of life for both older PLWH and their caregivers.

## Figures and Tables

**Table 1:** Semi-structured interview guide.

Dimension	Questions
Situation	In your daily life, have you ever experienced a strong awareness of your identity as a caregiver for someone living with HIV?
	Are there specific situations or contexts in which you feel concerned that others may discover your identity as a caregiver for someone with HIV?
	If others were to discover that you are providing care for an older adult with HIV, what would your immediate reaction be?
Cognition	Throughout your experience as a caregiver, how has your perception of HIV/AIDS evolved?
	Have you ever found yourself increasingly aware of issues related to HIV/AIDS the more you tried to ignore them?
Affect	Have you ever experienced differential treatment or discrimination due to caring for someone with HIV? If so, could you describe what occurred and how you responded to the situation?
Behavior	In your daily life as a caregiver, have you ever consciously modified your behavior or speech to prevent others from suspecting the patient’s HIV status?
	Since you began caring for the patient, to what extent, if any, have your social life and connections with friends and the community been affected?
	When you disclosed the patient’s condition to others, what thoughts or concerns were influencing your decision-making process?
Self-Evaluation	As a caregiver for a person living with HIV, how do you understand and interpret the responsibilities and implications of this role?
	When you encounter challenges in caregiving, what do you typically identify as the primary underlying causes?
	Throughout your caregiving experience, have you observed any impacts on your personal life or professional capabilities?

**Table 2:** Participant Demographic Characteristics (*N* = 15).

Characteristics	Frequency
Gender	
Male	5
Female	10
Age (in years)	
Range	27–75
Mean age	41.2
Highest level of education	
Primary school	3
Middle school	6
High school	2
University	4
Marital status	
Married	10
Unmarried	5
Caregiving duration (in years)	
≤1	1
1–5	5
≥5	9
Relationship with patients	
Daughter-in-law	1
Grandson	1
Parent	2
Adult child	11
Residence location	
Urban area	7
Rural region	8

**Table 3:** Themes and sub-themes extracted from the data.

Theme	Sub-Theme
Sources of affiliate stigma	Inadequate knowledge of HIV transmission routes
	Ageism
	Infidelity stigma
Experiences of affiliate stigma	Stigma endorsement
	Concealment of a family member’s HIV-positive status Psychological distress
Consequences of affiliate stigma	Estrangement among family members
	Substantial caregiver burden Social avoidance
Coping with affiliate stigma	Enhancing knowledge of HIV/AIDS
	Seeking social support

## Data Availability

The data are not publicly available due to privacy or ethical restrictions.
